# Integrating primary eye care with primary health care: tracing the journey

**Published:** 2022-03-01

**Authors:** Noopur Gupta, Souvik Manna, Suraj Singh Senjam, Vivek Gupta, Praveen Vashist

**Affiliations:** 1 Associate Professor, Ophthalmology: RP Centre, All India Institute of Medical Sciences, Delhi, India.; 2 PhD Scholar, Community Ophthalmology: RP Centre, All India Institute of Medical Sciences, Delhi, India.; 3 Additional Professor, Community Ophthalmology: RP Centre, All India Institute of Medical Sciences, Delhi, India.; 4 Associate Professor, Community Ophthalmology: RP Centre, All India Institute of Medical Sciences, Delhi, India.; 5 Professor, Community Ophthalmology: RP Centre, All India Institute of Medical Sciences, Delhi, India.

**Different international mandates recommend integrating primary eye care with primary health care to build a strong national, people-centred eye care programme.**

## The primacy of primary health care

In 1978, the World Health Organization (WHO) declared primary health care to be essential for achieving health for all. In 2018, Member States of the WHO strongly reaffirmed their commitment to primary health care.^1^ In its 1984 publication (and in the later edition in 1997), *Strategies for the Prevention of Blindness in National Programmes*, the WHO advocated implementing primary eye care through the primary health care system.^2^

We can define primary eye care as a ‘frontline’ activity, providing eye care and identifying disease before it becomes a serious medical issue.

Providing primary eye care is a challenge in many countries in WHO South-East Asia, as the health care system lacks adequate human resources. The government is the principal service provider at the primary level and predominantly manages eye care in India, Sri Lanka, Bhutan, and Maldives. Whereas in Nepal and Bangladesh, the NGO sector is the predominant service provider.^3^

## Global action on eye health

The ‘VISION 2020: the Right to Sight’ initiative launched in 1999 by the WHO and the International Agency for the Prevention of Blindness (IAPB) intensified advocacy efforts worldwide, strengthened national blindness prevention programmes, and supported national eye care plans. It focused initially on diseases that cause blindness and for which proven cost-effective interventions are available.^4^

Subsequently, the WHO global eye health action plan 2014–2019 for reducing avoidable visual impairment was ratified by Member States of the WHO, reaffirming their commitment to VISION 2020. This action plan has a broad vision, goal, and purpose. It specifies three objectives and specific actions to be taken by the Member States and international partners to enhance universal eye health, using the health systems approach.^5^

The *World Report on Vision* (2019) emphasised the need to make eye care an integral part of universal health coverage and incorporate integrated people-centred eye care (IPEC) in health systems as the strategy to achieve the same. The goal through IPEC is to assure a continuum of care against the spectrum of eye conditions throughout a person's life course, according to their needs.

Two new global targets were adopted by Member States of the WHO at the Seventy-fourth World Health Assembly in May 2021: to achieve a 40% increase in effective coverage of refractive error and a 30% increase in effective coverage of cataract surgical rate by 2030.^6^

## Role of community health workers in primary eye care

**Figure 1:**
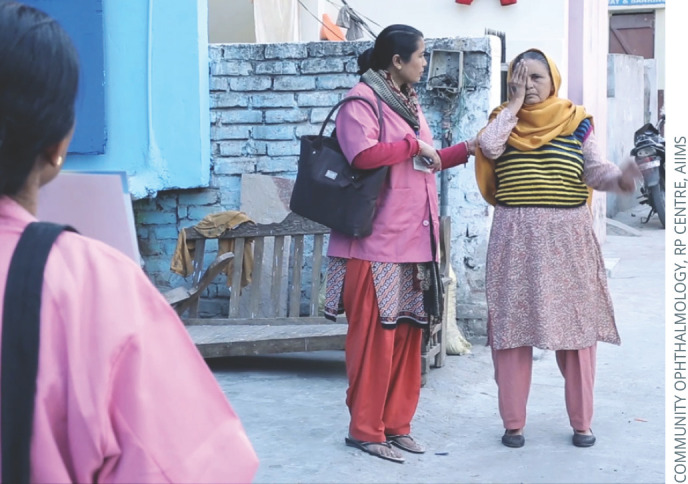
ASHAs assessing distance visual acuity in Delhi, using tumbling Snellen optotype. **INDIA**

Ever since the 1978 Alma Ata Declaration, there has been a growing interest in the role of community health workers as a crucial bridge between health facilities and communities. Among the successful community health worker programmes in the South Asia region are the accredited social health activist (ASHA) model in India (Figure 1) and the school eye health programme in Nepal. Primary eye care has been integrated entirely with primary health care in Bhutan and Sri Lanka by training the primary health care workers on primary eye care.

## Integration of eye care services within health care services at all levels

One of the major areas of focus for the global community is universal health coverage. Primary health care and integrated people-centred eye care are the necessary foundations for this effort. When sufficiently resourced, a primary health care facility can meet people's eye care needs throughout their life course. Such a facility can be used to raise awareness of the importance of maintaining eye health and adopting eye disease prevention behaviour, such as practising facial cleanliness to prevent active trachoma. In situations where more specialised services (e.g., cataract surgery) are required, primary care centres can facilitate referrals and coordination across providers and care settings.

To achieve a robust health care system that includes eye care, eye care should be integrated within the existing health care system at all levels. This means that eye care needs to be delivered in homes, schools, and other community settings; and integrated with inpatient and outpatient settings at the secondary and tertiary levels, improving service delivery, referral pathways, and accountability. Integration does not necessarily mean that everything is integrated into one package and delivered in one place. It means that services are so well connected that it becomes easy for users to navigate the health system and avail themselves of the services they need.

The continuum of care, which includes promotive, preventive, curative, rehabilitative and palliative services, would ideally be delivered in an integrated manner for all eye conditions afflicting individuals throughout their life. We feel integration in the true sense is to prevent eye care from functioning in a silo, and making it relevant to the broader goals of healthcare.
